# Validity and reliability of the Malay version of the PSC-12 scale among healthcare workers in Malaysia

**DOI:** 10.1371/journal.pone.0317744

**Published:** 2025-02-20

**Authors:** Norhasliza Abu Bakar, Awang Bulgiba, Marzuki Isahak

**Affiliations:** 1 University Putra Malaysia, Serdang, Malaysia; 2 Academy of Sciences Malaysia, Kuala Lumpur, Malaysia; 3 Academy of Occupational and Environmental Medicine Malaysia, Kuala Lumpur, Malaysia; Universiti Sains Malaysia—Kampus Kesihatan, MALAYSIA

## Abstract

The prime instrument used to measure Psychosocial Safety Climate (PSC) at the workplace is the PSC-12 scale questionnaire, which has been widely used by researchers worldwide. We set out to translate the PSC-12 scale into Malay and validate it among Malaysian healthcare workers, the first time this has ever been attempted. We carried out a cross-sectional study among 250 healthcare workers from 3 government health clinics and 15 identified clinical work units in a public hospital in the Klang Valley area, Malaysia. The English version of the PSC-12 scale was translated into the Malay language (Bahasa Malaysia) and back into English to check for content accuracy and validity. Psychometric properties of the questionnaire were assessed for internal consistency (Cronbach’s alpha coefficient), temporal stability for test-retest reliability (intra-class correlation) and construct validity (confirmatory factor analysis). The translated questionnaire had a persistently high content validity-index (CV-I) of 0.916. The hypothesised four-factor-structure model displayed high goodness of fit indices (RMSEA = 0.081, SRMR = 0.032, GFI = 0.919, AGFI = 0.869, CFI = 0.961, and TLI = 0.946), demonstrating good questionnaire construct validity. Each item subscale in the PSC-12 scale showed satisfactory internal consistency with Cronbach’s alpha (α) coefficient between 0.895 to 0.921. There was satisfactory temporal stability and test-retest reliability with ICC (2,1) for scores of 0.954 (total item score), 0.897 (Domain 1 score), 0.910 (Domain 2 score), 0.807 (Domain 3 score) and 0.806 (Domain 4 score) over the two-week interval. The Malay version of the PSC-12 scale is a valid and reliable instrument for use among healthcare workers in Malaysia as evidenced by its satisfactory psychometric measure and construct structure properties.

## Introduction

Work can directly influence the psychological health and well-being of workers [1]. Associations between work and psychological well-being are firmly established in the occupational stress literature through various causal relationships with some of these evidence now rooted in legislation [2,3]. Considered to be among the leading causes of workplace disability, mental health problems resulting from a poor psychological well-being is associated with low performance and reduced work productivity [4].

Psychosocial work factors have been defined as social characteristics of the work environment that interact with individual psychological factors [5]. An employed individual who stays in the workplace for at least 8 hours every day is exposed to psychosocial influence. This can be a psychosocial hazard. A psychosocial hazard is a major challenge in this context as it affects the mental well-being or mental health of the worker by overwhelming an individual’s coping mechanism and impacting the worker’s ability to work in a healthy and safe manner. It acts as a stressor to the development of work-stress related ill-health consequences. The term ‘psychosocial’ carries a broad meaning of interrelationship between the individual’s perception, behaviour and social environment but in the occupational health field it refers to a type of hazard exerted by the work and working environment [6]. A more comprehensive definition of ‘psychosocial hazard’ is given by the International Labour Organization (ILO) which is “the interactions between and among work environment, job content, organizational conditions and workers’ capacities, needs, culture, personal extra-job considerations that may, through perceptions and experience, influence health, work performance and job satisfaction” [7].

In comparison with physical hazards, which requires distinct measurements and whose impact depends on an individual’s susceptibility; psychosocial hazard is determined greatly by how the person perceives them (cognition appraisal) [8]. This explains why psychosocial hazards has never been imperatively significant to the ‘cause and effect’ in mental health problems, but it is also grossly erroneous to only believe psychosocial hazard is just relevant to mental health and to leave behind its implication on physical health outcomes [6]. The psychosocial hazard influence is also particularly revealing at the organizational or managerial level [9].

Psychosocial hazards at work are found in job design, the organization and management of work, and within the social environmental contexts of the workplace [10]. Multifactorial roles involved in the exposure of psychosocial hazards with the establishment of mental health consequences include personal factors and organizational factors. Previous studies in Australia, where the term psychosocial safety climate (PSC) was first proposed and developed, have shown that employee perceptions of low PSC were associated with a variety of adverse mental and physical health, as well as safety outcomes, such as psychological distress, emotional exhaustion, circulatory diseases, and occupational injuries [11–13]. Given that PSC is an upstream predictor of these outcomes, it is important to establish whether this construct is generalizable to different cultural settings, and to assess it in a variety of national contexts.

A healthy work environment brings safety to an employee’s physical and mental capabilities in performing his or her daily routine [14]. The work environment quality has been in the discourse ever since the Industrial Revolution when there was a transformation of economies from being based on agriculture and handicrafts to those based on large-scale industries, mechanized manufacturing, and the factory system. In order to understand the dynamics of the work environment climate and elements, researchers started to explore further on the facets underpinning a quantifiable work environment level known as the psychosocial safety climate.

PSC revolves around how organization or system factors contribute to the creation of psychosocial work characteristics [15]. It is a specific dimension of organizational climate that reflects employees’ shared perceptions on “policies, practices and procedures for the protection of workers psychological health and safety” [16]. PSC can be measured through a 12-item tool (PSC-12) that reflects the four main theoretical domains of PSC, i.e., (1) Management Support and Commitment for stress prevention through involvement and commitment, (2) Management Priority to psychological health and safety versus productivity goals, (3) Communication between the organization and employees on psychological health and safety issues, and (4) Organizational Participation and Involvement in protecting workers’ psychological health.

The first measurement scale developed by Gary B. Hall and Maureen F. Dollard in 2007 was based on the definition of PSC and it contained 26 items [17]. With successive large-scale, population-based research, the scale was systematically reduced to a parsimonious 12-item tool (PSC-12). The PSC-12 scale [17] consists of a four-factor scale with three items for each subscale. For the use of locals in particular countries, the PSC-12 scale has been translated into a few languages like Chinese [18], Japanese (PSC-12J) [19] as well as Swedish [20].

In view of the needs and demand for this questionnaire to be used in the Malaysian population, this study aimed to perform the translation and cultural adaptation of the original English version of the PSC-12 scale to a Malay version of the PSC-12 scale and then proceeded with the validation of the translated Malay version PSC-12 scale. To our knowledge, there has been no comparable validation study published for the Malay version of the PSC-12 scale conducted among healthcare workers in Malaysia. Hence, this is the very first validation study of the PSC-12 scale in the Malay language in the aforementioned population.

## Materials and methods

### Study designs and participants

This cross-sectional study was carried out over 3 months duration from July to September, 2014 among 250 healthcare workers from 3 government health clinics and 15 identified clinical work unit in a public hospital in the Klang Valley area, Malaysia.

The general guideline on the adequate sample size for factor analysis includes Tabachnick’s rule of thumb that suggests having at least 300 cases [21], but another work by Comrey and Lee [22] in their guide stated the sample sizes: 100 as poor, 200 as fair, 300 as good, 500 as very good, and 1,000 or more as excellent. To accommodate this and based on the feasibility of the setting and procedure, a sample of 200 participants was determined for the purpose of factor analysis assessment in this current research. The selection of the multicentre healthcare facility was to provide considerable generalization of the validation study across the rural-urban setting of healthcare. Ethics approval was granted by the University of Malaya Medical Centre Medical Ethics Committee (MEC 201311-0538) for this study. Written informed consent obtained from each individual participants who enrolled in the study to meet the ethical conduct of research with human subject.

### Materials and procedure

#### Translation and cultural adaptation of Malays version PSC-12 scale.

The translation process of the original English version of the PSC-12 scale involved a concurrent forward-backward translation by both a medical expert and a language expert. The goal of translation was to prepare a survey in the Malay language that allowed for the intended original meaning of the questionnaire to come across and which was also grammatically sound for use among Malay-speaking healthcare workers.

After the reconcilement of the forward and backward translated Malay version of the PSC-12 scale, a sentence revision was carried out by expert panel members to check for content validity. It included the content applicability of each expressed item with the local culture and also the phrasing clarity on the comprehension of each sentence in the questionnaire. The translated Malay version of the PSC-12 scale was sent to a clinical psychologist (majoring in Industrial and Organizational psychology) and a public health physician (subspecialising in Occupational Health). The expert panel rated each of the translated items for clarity and applicability based on a four-point Likert scale (1 = not relevant, 2 = somewhat relevant, 3 = quite relevant, 4 = highly relevant). The CV-I (Content Validity Index) was calculated to quantify the degree of the experts’ agreement regarding the content relevance of what the instrument is proposed for. The index was calculated based on the number of items that the expert rated above 3 on the four-point scale. Scholar agreement on the acceptability Item CV-I is 1.00 for ‘five or fewer experts’, meaning that all experts must agree or rate above 3 for each item content validity [23]. The content validity assessment was conducted by two experts panel due to resource constraints in current study. While this approach provided valuable insights, we acknowledge the potential bias associated with a limited number of evaluators and recommend including additional experts in future studies.

**Fig 1:**
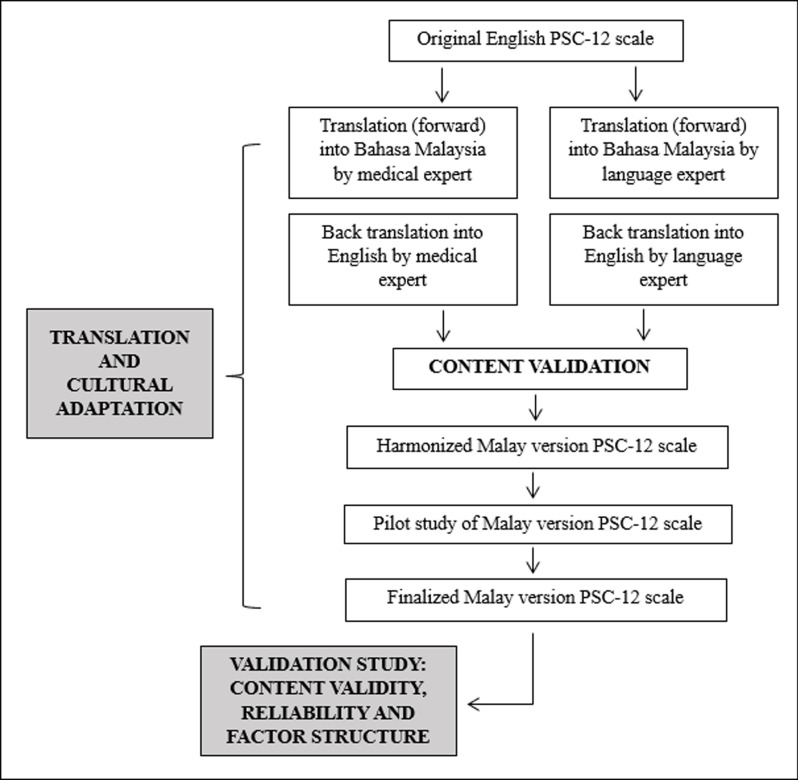
Summary of translation, cultural adaptation and questionnaire validation study of Malay version PSC-12 scale.

Finally, a harmonized Malay version of the PSC-12 scale was produced that was satisfactory in terms of cultural suitability and linguistic fluency. A pilot test was then performed upon completion of the translation to identify any flaws in the harmonized version of the Malay PSC-12 scale which might affect the comprehension of the subjects. The entire process is summarized in Fig 1.

#### The psychometric assessment (validity and reliability) of Malay version PSC-12 scale.

The finalized version of the Malay version of the PSC-12 scale was then tested for its psychometric properties including reliability and validity. In detail, the assessed elements during this stage was **internal consistency** (Cronbach’s alpha coefficient), **temporal stability** (intra-class correlation coefficient [ICC]), **factor structure** (confirmatory factor analysis) and test-retest agreement which is on the **reliability** of the tested instrument.

With the cooperation with the Team Leader (Medical Officer/ Paramedic) at the respective health clinics and clinical work units, we arranged a briefing session for potential healthcare worker participants. During the sessions, information leaflets and informed consent forms were distributed. Interested and volunteered healthcare worker submitted their consent forms at the end of the briefing sessions and received the self-administered set of questionnaires which consisted of two parts of the study instrument (see Table 1). Two sessions of data collection were carried out at 2-week intervals to allow test-retest assessment (reliability) using a similar set of questionnaires.

**Table 1:** Instrument used in validation and reliability study of Malay version PSC-12 scale.

Instrument tested	Domain interested	Evaluation method
A self-report questionnaire	Socio-demographic and occupational characteristics	Description of participants’ age, gender, ethnicity, marital status, education level, occupation
Malay Version PSC-12 Scale	Psychosocial environment at the workplace	Internal consistency, temporal stability (test-retest reliability), factor structure (construct validity)

## Statistical analysis

Data entry and analysis were done using IBM SPSS® Statistics version 21.0 [24]. Data coding, entry and checking were carried out vigilantly to get rid of false data entries and missing values, including double data entry by the researcher.

Descriptive statistics were used to describe the socio-demographic characteristics of the participants enrolled in the validation study including mean, standard deviation, range and frequencies. The content validity of the translated questionnaire was assessed via content validity index (CV-I) score with acceptable CV-I scores of at least 0.8 for two expert panel [25,26]. To examine the structural validity, confirmatory factor analysis (CFA) was carried out, which allowed the researcher to assess the goodness of fit for the structure of the PSC-12 scale in other literature (i.e., four factor structure). Model fit was assessed using a combination of fit indices including the goodness of fit index (GFI), the adjusted goodness of fit index (AGFI), the comparative fit index (CFI), the Tucker–Lewis index (TLI) to quantify the relative distinctiveness between the data and estimated values obtained from the model of the questionnaire. Values of more than 0.90 were considered to be acceptable. The root mean square error of approximation (RMSEA) with cut-off point of <0.08 was also computed to measure the discrepancy of the model used in data collection.

To examine internal consistency, Cronbach’s alpha coefficient was calculated with the assessed set of items (intercorrelation) considered to be acceptably reliable to measure a similar construct when Cronbach’s alpha ≥ 0.70. Finally, parametric statistics for test-retest reliability, intra-class correlation coefficient (ICC [2,1]) were calculated to assess the temporal stability of the instruments’ items. ICC values ≥ 0.70 were considered as acceptable levels of test-retest reliability for the four domains of the PSC-12 scale.

## Results

### Socio-demographic profiles

Participants of Group 1 (healthcare workers in health clinics) had a mean age of 32.78 (SD ± 7.768) years and were predominantly female staff (90%) with a mean duration of service at the current workplace of 2.48 (SD ± 0.995) years. Group 2 participants (healthcare workers in hospital clinical work units) were also predominantly female (79.5%) with a mean age of 30.31 (SD ± 8.172) years, and a mean duration of service at the current workplace of 6.09 (SD ± 6.310) years. The majority of respondents were of Malay ethnicity (>80.0% for both groups) and >50.0% of them had attained a diploma as their highest educational achievement. The enrolled study participants were from various healthcare worker categories including doctors, pharmacists, paramedics (nurses and medical assistants), physiotherapists, laboratory technicians, health attendants as well non-clinical staff. The documented socio-demographic profiles are summarized in Table 2.

**Table 2:** Socio-demography profile of study participants.

Variables	Group 1 (n = 50)		Group 2 (n = 219)	
	Number (%)	Mean (SD)	Number (%)	Mean (SD)
**Age**	–	32.78 (7.768)	–	30.31 (8.172)
**Gender**				
Male	5 (10)	–	44 (20)	–
Female	45 (90)	–	174 (79.5)	–
**Ethnicity**				
Malay	41 (82)	–	205 (93.2)	–
Chinese	2 (4)	–	6 (2.7)	–
India	7 (14)	–	7 (3.2)	–
Others	–	–	1 (0.5)	–
**Education status**				
Secondary	7 (14)	–	55 (25)	–
Tertiary				
Diploma	29 (58)	–	150 (68.2)	–
Degree	14 (28)	–	13 (5.9)	–
Master	–	–	1 (0.5)	–
**Types of job**				
Medical officer	9 (18)	–	3 (1.4)	–
Houseman	–	–	3 (1.4)	–
Pharmacist	3 (6)	–	–	–
Physiotherapy	–	–	1 (0.5)	–
Staff nurse	27 (54)	–	150 (68.2)	–
Medical assistant	3 (6)	–	10 (4.5)	–
Laboratory tech.	1 (2)	–	–	–
Health attendant	5 (10)	–	32 (14.5)	–
Driver	–	–	8 (3.6)	–
Clerk	2 (4)	–	12 (5.5)	–
**Duration of service at current work place (in years)**	–	2.48 (0.995)	–	6.09 (6.310)

### Modified items in Malay version PSC-12 scale

During the translation and adaptation process which involved face validation in a focus group, one of the translated items sentences and two item wordings were further corrected and reviewed. The finalised version of the Malay PSC-12 scale was modified without removing any of the sentences or item wordings of the original English version PSC-12 scale questionnaire. The modified phrases in Malay language are as follows:

*7. … ‘Terdapat komunikasi yang bagus tentang isu keselamatan psikologi yang memberi kesan kepada saya’*

*9. … ‘Sumbangan saya untuk menyelesaikan masalah kesihatan dan keselamatan pekerjaan dalam organisasi adalah di ambilkira’*

### Content validity assessment of Malay version PSC-12 scale

The content validity was assessed by two experts involved in this study using content validity index (CV-I) scores. Both persons rated with consistently high markings, which was a 3 or 4 on the Likert scale scores for each item in the PSC-12 scale questionnaire, as the elements accomplished its relevance to measure the representative construct. The summary of the CV-I scoring is in Table 3.

**Table 3:** Summary of Content Validity Index (CV-I) scores by expert panel.

Item number	Items	Items rated 3 or 4 points on Likert’s scale score		Items CV-I
		Expert 1	Expert 2	
1	***Pihak pengurusan di tempat saya bekerja bertindak pantas dalam menyelesaikan masalah/isu yang memberi kesan kepada kesihatan psikologi pekerja.*** The management in my workplace acts quickly in resolving problem/issue that affects psychological health of employees.	√	√	1
2	***Pihak pengurusan bertindak tegas apabila isu berkenaan status psikologi pekerja dibangkitkan.*** The management acts decisively when an issue about psychological status of employees is raised.	√	√	1
3	***Pihak pengurusan menunjukkan sokongan untuk pencegahan stress/tekanan melalui penglibatan dan komitmen.*** The management shows support for stress prevention through involvement and commitment.	√	√	1
4	***Kesejahteraan psikologi pekerja menjadi keutamaan bagi organisasi ini.*** Psychological well-being of employees is a priority for this organization.	√	√	1
5	***Pihak pengurusan dengan jelas menganggap kesihatan psikologi pekerja adalah sangat penting.*** The management clearly considers the psychological health of employees to be of great importance.	√	√	1
6	***Pihak pengurusan dengan jelas menganggap kesihatan psikologi pekerja adalah sama penting dengan produktiviti.*** The management clearly considers psychological health of employees to be as important as productivity.	√	√	1
7	***Terdapat komunikasi yang bagus tentang isu keselamatan psikologi yang memberi kesan kepada saya.*** There is good communication about psychological safety issues in this organization which affect me.	√	√	1
8	***Pengurus/penyelia saya sering memberi maklumat berkenaan kesejahteraan psikologi di tempat kerja.*** My manager/supervisor often provides information on psychological well-being in the workplace.	–	√	0.5
9	***Sumbangan saya untuk menyelesaikan masalah kesihatan dan keselamatan pekerjaan dalam organisasi adalah di ambilkira.*** My contributions in resolving occupational health and safety problem in the organization are listened.	√	–	0.5
10	***Penglibatan dan konsultasi dalam kesihatan dan keselamatan psikologi di tempat kerja saya dijalankan bersama pekerja, kesatuan dan wakil kesihatan dan keselamatan.*** Participation and consultation in psychological health and safety involves the employees’, unions and health and safety representatives in my workplace.	√	√	1
11	***Pekerja digalakkan untuk melibatkan diri dalam isu berkenaan keselamatan dan kesihatan psikologi.*** Employees are encouraged to involve in psychological safety and health matters.	√	√	1
12	***Pencegahan stress/tekanan dalam organisasi saya melibatkan semua pihak.*** The prevention of stress in my organization involving all parties.	√	√	1
	**Total CV-I scores**	**11**	**11**	**11**
	**Average CV-I scores**	**0.916**	**0.916**	**0.916**

CV-I = Content Validity Index.

### Construct validity (factor structure) assessment of Malay version PSC-12 scale

**Fig 2:**
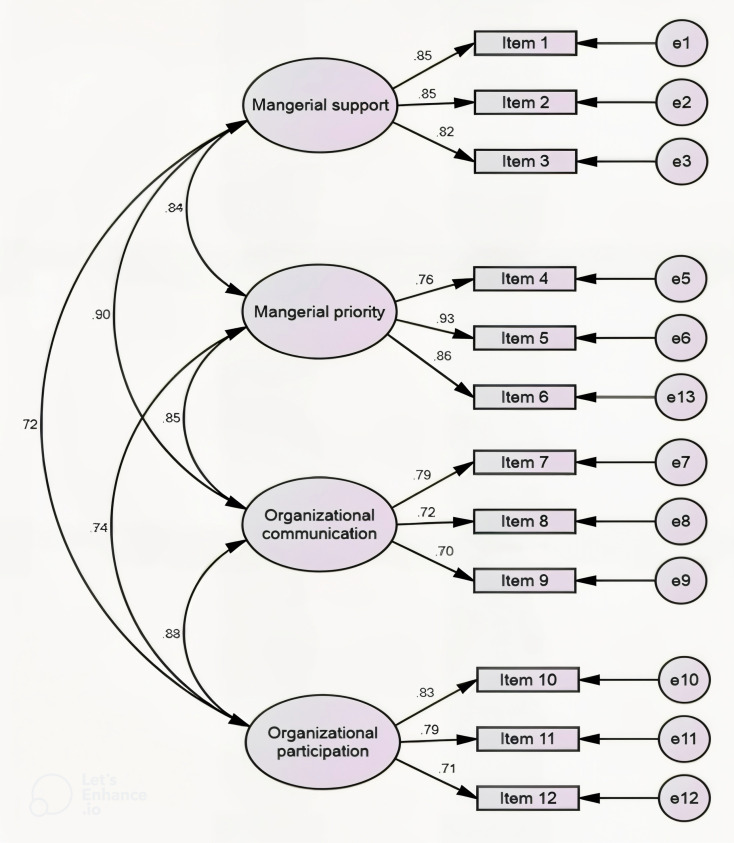
Confirmatory Factor Analysis (CFA) of PSC-12 scale four factor structure.

The hypothesised four-factor-structure model of PSC-12 analysis reported better goodness of fit indices; RMSEA = 0.081, SRMR = 0.032, GFI = 0.919, AGFI = 0.869, CFI = 0.961, and TLI = 0.946. The results show that the hypothesised four-factor structure model of the PSC-12 scale is a satisfactory and fit model. The analysed result is summarized in Table 4 and Fig 2.

**Table 4:** Goodness of fit indices for Confirmatory Factor Analysis (CFA) of PSC-12 scale.

Model	χ²	GFI	AGFI	TLI	CFI	RMSEA	SRMR
**Model 1: One factor**	5.046	0.822	0.745	0.851	0.884	0.142	0.046
**Model 2: Two factor**	2.458	0.923	0.788	0.954	0.962	0.084	0.040
**Model 3: Four factor**	2.447	0.919	0.869	0.946	.961	0.081	0.032

GFI = Goodness of fit index, AGFI = Adjusted goodness of fit index, TLI = Tucker Lewis Index, CFI = Comparative fit index, RMSEA = Root mean square error of approximation, SRMR = Standardized root mean square residual.

### Internal consistency assessment of Malay version PSC-12 scale

For the PSC-12 scale, each item subscale measure showed good and satisfactory internal consistency with Cronbach’s alpha (α) coefficient between 0.895 to 0.921. All the twelve PSC scale items demonstrated moderate to high corrected item – total correlation value (0.461–0.772). Table 5 summarizes the results for the internal consistency assessment.

**Table 5:** Cronbach’s alpha (α) coefficient for each item measure of Malay version PSC-12 scale (n = 50).

PSC items	Corrected item-total correlation	Cronbach’s alpha (α) if item deleted
1	0.670	0.900
2	0.633	0.902
3	0.772	0.896
4	0.729	0.898
5	0.730	0.898
6	0.800	0.895
7	0.461	0.916
8	0.770	0.896
9	0.736	0.899
10	0.750	0.898
11	0.749	0.899
12	0.502	0.921

### Test-retest reliability and temporal stability assessment of Malay version PSC-12 scale

The test-retest reliability of the PSC-12 scale was done at two-week intervals with ICC (2,1) for total items score of 0.954, Domain 1 = 0.897, Domain 2 = 0.910, Domain 3 = 0.807 and Domain 4 = 0.806. These results suggest a satisfactory and adequate measure of the PSC-12 scale’s temporal stability or reliability. Details of the results are summarized in the following Table 6.

**Table 6:** PSC-12 scale ICC (Intra-cluster Correlation) reliability index result.

Items	ICC (2,1) value	95% CI	
		Lower border	Upper border
Total items PSC score	0.954	0.932	0.972
Domain 1	0.897	0.840	0.939
Domain 2	0.910	0.861	0.947
Domain 3	0.807	0.700	0.885
Domain 4	0.806	0.698	0.884

*Domain 1 = Management support and commitment for stress prevention.

*Domain 2 = Management priority for psychological health and safety.

*Domain 3 = Organizational communication.

*Domain 4 = Organizational participation in psychological health and safety.

## Discussion

The PSC-12 scale is an established 12-item four-factor substructure instrument to measure employee perception of PSC [17]. In the preliminary stage, a focus group face validation performed among a group of healthcare workers after the forward-backward translation of the English version of the PSC-12 scale to the Malay language resulted in minor modifications to the sentences in items 7 and 9 of the PSC-12 scale. This has resulted in a translated Malay version of the PSC-12 scale which is comprehensible and safe to be used in the Malaysian context. This is attested to by a satisfactory Content Validity Index (CV-I) rated by two experts (average CV-I scores: 0.916).

The English version PSC-12 scale remains the main tool used to measure PSC, along with the few different language versions reported in the previous literature [18–20], but for the 300 million Malay-speaking peoples of South-East Asia, there is no standard version for them. The construct validity of this Malay version of the PSC-12 scale used in the current research reported that all the 12-items in the four-factor structure (goodness of fit index, GFI: 0.919) fitted the data better than the one-factor structure (GFI: 0.822), which was reported in the development of the original PSC-12 scale [17]. This ensured that the measured construct of interest in the Malay version of the PSC–12 scale fitted the four-factor structure in assessing the organizational dimension of the psychosocial work environment. However, the translated Malay version of the PSC-12 scale was only tested among healthcare workers as the main study subject in the current research. Therefore, no multigroup analysis was performed across other different groups of occupation, and this Malay version of the PSC-12 scale is a valid measure of psychosocial work environment specifically among healthcare workers. In addition, this study also did not include smoking and alcohol consumption as assessment criteria, as these variables were outside the primary scope of validating the PSC-12 scale. However, we acknowledge their relevance in psychosocial evaluations and recommend future studies incorporate these factors to enhance the comprehensiveness of the scale.

There is no doubting the importance of a valid questionnaire that is reliable and can produce consistent results in repeated trials [27]. We therefore believe that this Malay version of the PSC-12 scale, which has been rigorously validated in a diverse sample, will enable other researchers using it to be confident of its consistency and reliability when using it in future research measuring similar constructs [28]. It adds richness to occupational health research by adding another important tool to assess the psychosocial safety climate in the workplace. Whilst the majority of readily available Malay language translated questionnaires measure physical hazards at the workplace, the recently validated Malay version of the PSC-12 provides a set of contemporary tools to assess the spectrum of psychosocial hazards in the workplace. This will be especially valuable when one wishes to study these parameters at the organizational level. We believe that it has the potential to assist occupational health interventions at the managerial level which in turn can lead to valuable policy changes for workers.

## Conclusion

This Malay version of the PSC-12 scale is valid and highly reliable and can be used with great confidence among Malay-speaking healthcare workers. Given the diverse sample used in this validation and the comprehensiveness of the validation, we believe that this tool can be used to measure PSC in any workplace with large numbers of Malay-speaking workers.
